# 4D Printing of Magnetically Responsive Materials and Their Applications

**DOI:** 10.34133/research.0847

**Published:** 2025-09-22

**Authors:** Yunfang Bai, Siying Zhu, Jin Liang, Ruizhe Xing, Jie Kong

**Affiliations:** ^1^ MOE Key Lab of Materials Physics and Chemistry in Extraordinary Conditions, Shaanxi Key Lab of Macromolecular Science and Technology, School of Chemistry and Chemical Engineering, Northwestern Polytechnical University, Xi’an 710072, P.R. China.; ^2^ Research & Development Institute of Northwestern Polytechnical University in Shenzhen, Shenzhen 518063, P.R. China.

## Abstract

In the field of 4-dimensional (4D) printing, magnetically responsive smart materials have attracted much attention due to their advantages such as large deformation rate, fast dynamic response, and remote controllability. Focusing on this field, this review systematically reviews the research progress of magnetically responsive 4D printing technology from 3 dimensions: printing process, material design, and interdisciplinary applications. Firstly, it elaborates on the core principles and key technologies of 4D printing and conducts an in-depth analysis of the design principles, response mechanisms, and performance optimization strategies of magnetic shape memory materials, providing a clear framework for understanding the fundamental principles of this technology. On this basis, it explores the innovative applications of magnetically responsive 4D printing in the fields of biomedical tissue engineering, intelligent robotics, and functional devices, highlighting its substantial advantages in non-contact manipulation, high-precision response, and biocompatibility, and demonstrating the practical application value of the technology. Finally, it proposes a collaborative design framework of “material–process–application” in order to promote the application of magnetically responsive materials in interdisciplinary fields.

## Introduction

The concept of “4-dimensional (4D) printing” was first officially proposed by Professor Skylar Tibbits and his team at the Massachusetts Institute of Technology in 2013. Initially, it referred to a new technology that allows the shape of 3-dimensional (3D)-printed objects to change over time [1–3]. With the development of technology, 4D printing has evolved into a technology that demonstrates the capabilities of additive manufacturing structures. Under external stimuli such as magnetic fields, gases, light, and heat, its shape, properties, or functions can undergo controllable changes over time [4–7]. The aforementioned stimulus–response mechanisms have completed conceptual verification studies in interdisciplinary fields such as aerospace engineering and biomedical engineering, and have shown practical application potential.

The self-healing ability, adaptivity, and self-assembly properties of 4D-printed smart materials represent their core technical advantages. The self-healing property endows materials with the ability to autonomously restore their structural and functional integrity after being damaged, substantially improving the service life and reliability of devices [8]. Adaptivity is manifested in the ability of materials to make real-time adjustments to their physical or chemical properties according to dynamic variations in external environmental parameters (such as temperature, humidity, and stress fields). The self-assembly property provides an effective approach for the efficient fabrication of complex 3D architectures, which is usually achieved through pre-programmed material responses or guidance by external stimuli [9]. This set of synergistic effects makes 4D printing technology a practical method for constructing controllable dynamic environments and intelligent responsive systems [10].

**Fig. 1.**
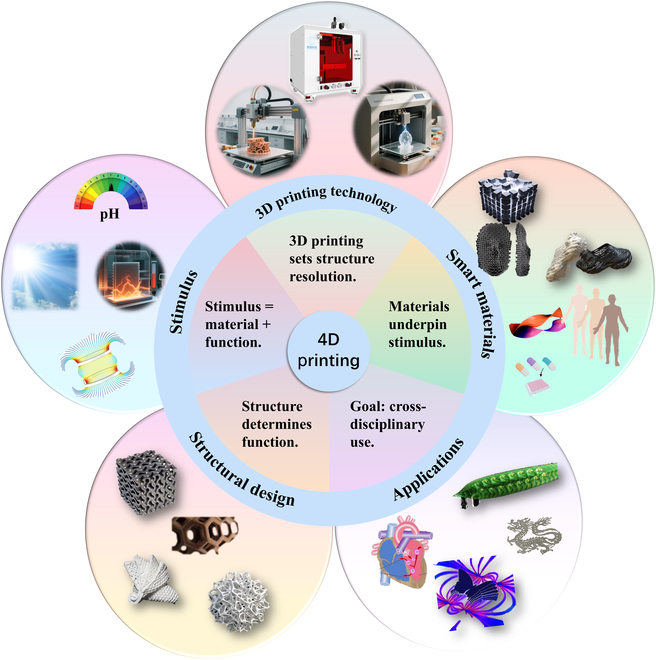
General overview of 4D printing.

Currently, the main external stimuli that drive time-dependent shape morphing or functional switching of 4D-printed structures include thermal, optical, aqueous environments (e.g., water, solvents, pH, and ions), mechanical force, magnetic field, and electric field [11–13]. Among them, thermal actuation is widely used in the fields of soft actuators and soft robotics due to its ease of implementation and precise controllability. In contrast, magnetic actuation, with its advantages of non-contact and deep-tissue penetration capability in manipulation, shows great potential in fields such as biomedical engineering and smart devices [14–16]. Figure 1 illustrates the basic principles of 4D printing, clearly showing the technological evolution and broad application prospects achieved on the basis of traditional 3D printing through the dual roles of smart material design and stimulus–response mechanisms.

This review systematically elaborates on the latest research progress of magnetically responsive smart materials for 4D printing. Magnetically responsive materials are a type of smart materials with the characteristic of magnetically induced strain [17]. Their core mechanism is to realize magnetic-to-mechanical energy conversion through the force-induced effect or magneto-strictive effect of internal magneto-sensitive particles in the magnetic field, thereby achieving precise and controllable deformation response [18–20]. Under the stimulation of various magnetic field modes (such as static magnetic field, alternating magnetic field, and gradient magnetic field), this type of materials all exhibit stable response performance. Their salient features are mainly reflected in the following aspects: excellent mechanical response performance enables large-angle bending or complex deformation; ultrafast response speed (millisecond-scale) meets the requirements of dynamic manipulation; high electromechanical conversion efficiency can reduce energy loss; and the unique non-contact remote manipulation capability makes them suitable for operations in confined or hazardous environment operations [21].

However, magnetically responsive smart materials still have many limitations: the materials lack reprogramming capability, and it is difficult to change their response mode after forming; the printed structure is limited by the precision of existing additive manufacturing equipment, and its complexity needs to be improved; the types of printable materials are few, mainly concentrated in polymer-based composites, and the printing technology of metal-based or ceramic-based magnetically responsive materials is not yet mature [22–24]. In recent years, with the advancement of nanocomposite technology and 3D printing processes, research on magnetically responsive smart materials has increasingly increased. Through approaches such as optimizing the dispersibility of magneto-sensitive particles and improving the interface bonding performance of materials, the above-mentioned defects are gradually being overcome [19,25,26]. At present, such materials have been widely used in the biomedical field, robotics field, and flexible electronics field [27–29].

This review provides a comprehensive review of the basic aspects of magnetically responsive materials. The “Printing Technology” section compares the applicability of mainstream printing technologies direct ink writing (DIW) and stereolithography (SLA)/digital light processing (DLP), analyzes the specific requirements of these technologies for materials, and discusses the key factors affecting printed components. The “Magnetically Responsive Materials” section examines the composition system of magnetically responsive materials and elaborates on their magneto-strictive deformation principles, performance parameter testing, and optimization strategies. The “Applications of 4D Printing Magnetically Responsive Materials” section sorts out the application cases and prospects of magnetically responsive materials in multiple fields, including biomedicine, robotics, and intelligent structures and devices. This review presents the current research status from multiple dimensions, providing comprehensive and in-depth references for researchers in related fields.

## Printing Technology

3D printing technology is a digital model-based layer-by-layer manufacturing technology, which directly shapes 3D solid components by accurately stacking materials layer by layer through computer control, also known as additive manufacturing [30]. ISO/ASTM52900-15 defines 7 categories of additive manufacturing processes: material extrusion, vat photopolymerization, powder bed melting, material jetting, binder jetting, sheet lamination, and directed energy deposition [31]. 4D printing is an additive manufacturing technology developed in recent years, which adds the dimension of “time” to 3D printing, so that the printed object has new functions such as “self-recovery, self-perception, self-assembly, and self-deformation” [32]. 4D printing technology uses 3D printer equipment, specially adapted during layer-by-layer processing, using stimulus-responsive materials to meet the different ongoing needs of different fields. At present, the additive manufacturing process of 4D printing has been broadly classified as liquid coagulation, powder coagulation, and direct material extrusion by its related printing mechanisms [33]. In the following section, we will introduce the production routes, technical characteristics, product raw materials, and related application research of magnetically responsive materials currently using this technology for the 4 production processes of light curing, fused deposition modeling (FDM), DIW, and selective laser sintering (SLS). The basic information of these 4 printing technologies is summarized in Table 1.

**Table 1.** Comparison of printing technologies

Printing technology	Applicable materials	Impact parameters	Advantages	Limitations	Applications	Reference
DIW	Polymer, metal, ceramic composites	Ink rheology, temperature, print speed, nozzle diameter	Wide range of materials, multimaterial printing, precise control of nanofillers	Highly demanding ink rheology, curing speed affects structural stability	Sensors, capacitors, soft robot structures	[200–205]
SLS	Polymers, metal powders	Powder particle size, temperature, sweep speed, laser beam wavelength	High mechanical strength without support structure	High powder fluidity requirements, complex post-processing	Medical scaffolds, flexible electronics	[206–208]
SLA/DLP	Photosensitive resin, hydrogel	Wavelength, spot size (SLA), projector resolution (DLP)	Ultra-high precision, smooth surfaces, fast print speeds	Brittle materials, difficult-to-process composites	Flexible electronics, electromagnetic shielding materials	[209–213]
FDM	Thermoplastic polymers	Nozzle diameter and temperature, bed height and temperature, print speed	Simple operation, low cost, easy material modification	Weak interlayer bonding, rough surface, limited precision	Drug-carrying materials, multifunctional sensing, photothermal applications	[214–219]

### Direct ink writing

**Fig. 2.**
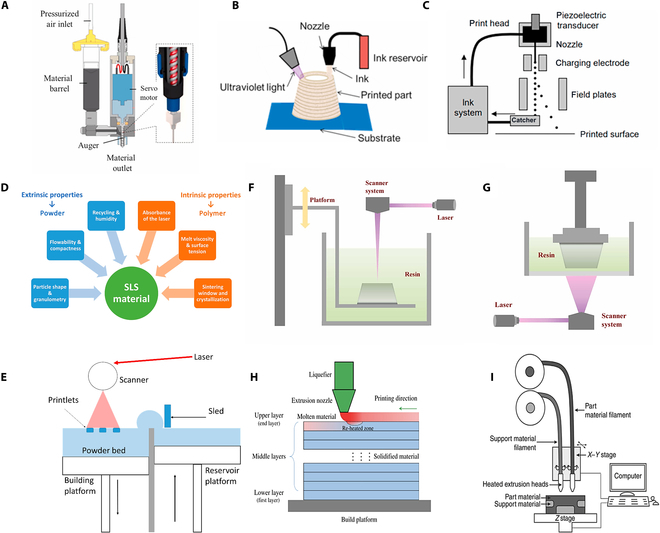
Schematic diagram of different types of DIW extruders: (A) screw injection, (B) pneumatic, (C) electric piston [37]. Copyright 2021, Elsevier. (D) Schema of the SLS printer [198]. Copyright 2021, MDPI. (E) Characterization requirements for SLS printed polymer powders. (F) Stereolithography printing schematic. (G) Digital light processing printing schematic [199]. Copyright 2024, Elsevier. (H) Schematic cross-section of a 3D structure printed using FDM. (I) Schematic of the general equipment for FDM [30]. Copyright 2020, MDPI.

DIW is an additive manufacturing technology based on the principle of micro-extrusion; firstly, the virtual model of the printed object is designed on the computer, including its size and shape, and then the printer extrudes the functional ink through the precision nozzle by the pressure drive, and at the same time, it realizes the precise positioning of the 3D motion trajectory under the control of the computer, and finally molds the complex 3D structure through the layer-by-layer stacking method [34,35]. Pneumatic, mechanical, and solenoid-based extrusion systems are the 3 most common types of DIW extruders currently studied (Fig. 2A to C) are schematic diagrams of the 3 extrusion systems) [36,37]. High print resolution can be achieved by employing micronozzles in DIW, which is particularly advantageous for radio frequency and self-powered microdevices [10]. When using DIW printing technology, the performance of the ink largely determines the performance and shape of the printed object. In order to realize high-speed, reliable, and high-precision mass manufacturing, DIW inks should have good viscoelastic and shear–stress properties, and satisfy the conditions of precise extrusion, instant shape retention, strong structural support, fast and defect-free curing, perfect interlayer fusion, material homogeneity, controllable curing time, as well as reproducibility and stability compatible with mass production [38–41]. Compared with other printing methods, DIW has unique advantages: it has wide material adaptability and less consumables and supports multimaterial printing, which is especially suitable for the preparation of nanocomposites in the laboratory environment, and it can precisely regulate the distribution and structure of nanofillers with different contents (including transparent and opaque systems) [42,43]. At present, using DIW technology, various types of materials have been successfully printed, such as metal particles, polymers, ceramics, and other materials [44–49]. In ceramic printing, Cárcamo-Gutiérrez et al. [50] developed 3D-printable ZnO nanoparticle-based ceramic pastes free of organic binders. They employed a solvent–co-solvent system of deionized water and ethylene glycol, with silica nanoparticles as inorganic binders, and optimized the formulation by adjusting the volumetric ratio of water to ethylene glycol and the content of silica nanoparticles to achieve suitable rheological properties for DIW. Focusing on another ceramic system, Chen et al. [51] explored polymer-derived ceramics (PDCs) for phosphor thin-film sensors. They prepared a PDCs-Y_2_O_3_:Eu phosphor-ceramic precursor mixture by mixing a ceramic precursor solution with Y_2_O_3_:Eu powder, then applied it via DIW, followed by high-temperature sintering to achieve ceramization, offering a distinct strategy for forming stable ceramic structures through precursor pyrolysis. Turning to metal materials, Mo et al. [52] utilized a camphene-based metallic ink for DIW of titanium scaffolds. Camphene served as the solvent, with polyethylene as the binder and Hypermer KD-4 as the dispersant; the ink was extruded layer by layer, and after treatment including camphene removal and sintering, hierarchical porous structures were formed, illustrating how organic solvents can tailor metal ink properties for DIW. In line with the additive optimization approach seen in the above studies, Krisnadi et al. [53] improved the printability of liquid metal foams (LMFs) by incorporating glycerol and tannic acid. These additives modified the rheology and stability of LMFs, preventing phase separation during extrusion and enabling more uniform DIW printing, highlighting the universal role of additive adjustment in enhancing DIW performance across material types.

### Light curing

Light curing molding technology utilizes an ultraviolet light source. By precisely controlling its wavelength and intensity, the focused light beam scans point by point over the surface of the photosensitive resin and induces curing. This process first completes single-layer molding in a point-line-plane sequence. Subsequently, the worktable moves precisely by one layer thickness in the vertical direction, and the steps of scanning and curing are repeated layer by layer in sequence, ultimately accumulating to construct a complete three-dimensional entity. Depending on the differences in control systems, light-cured 3D printing technologies cover the following main types: SLA, DLP, continuous liquid interface generation, 2-photon polymerization, and high-area rapid printing. This article describes SLA and DLP, the working schematic diagram of which is shown in Fig. 2F and G [22,54–56]. SLA technology uses a 355-nm laser beam to achieve precision molding through top-down or bottom-up photopolymerization reactions. The core process is as follows: the molding platform is initially immersed in a photosensitive resin tank, so that the liquid surface and the platform spacing maintain the thickness of a single layer; the laser beam according to the 3D model slice data accurately scans the resin surface, inducing the liquid resin layer-by-layer curing; each layer of the cross-section molding is completed, the platform is lowered by one layer thickness in the vertical direction, and the process continues with scanning and curing the next layer, ultimately building up a high-precision three-dimensional entity. The thickness of a single layer can range from 12 to 150 μm. In practice, a thickness of 100 μm is typically chosen. Standard SLA printers print at speeds in the range of 10 to 20 mm/h. Because the laser beam can move over a large space, SLA technology can be used to print large parts and the accuracy produced by this technology is related to the diameter of the laser beam at the curing point (spot size) [57,58]. Unlike laser progressive scanning of SLA, DLP technology cures the entire input slice layer at once and maintains the resin surface used during the printing process in constant contact with the platform, thus guaranteeing the precision, resolution, and quality of the print [59]. These 2 technologies can realize commercial and laboratory-made materials, which have the advantages of fast printing speed, high printing resolution, and smooth surface of the printed object, and can prepare workpieces with high dimensional accuracy requirements [60,61]. However, because SLA technology utilizes a focused laser beam for point-by-point curing on the machining plane, its molding accuracy is largely dependent on the laser spot diameter, whereas the accuracy of DLP technology is limited by the pixel-matrix resolution of its projection system; the use of SLA technology is usually able to produce parts with higher accuracy and better mechanical properties. However, due to the limitations of light-curing optical requirements, they are only suitable for light-curing resins, making it difficult to construct composites with opaque fillers [62,63]. Due to the brittleness, deformation, poor weather resistance, and low biocompatibility of light-curable materials, light-curable printing technologies and materials are currently mostly used in the field of temporary alternative materials, such as dental restoration, orthodontics, dental surgery, models, and molds [57].

### Selective laser sintering

Powder bed fusion, one of the 7 classifications of 3D printing recognized by the American Society for Testing and Materials, centers on the selective solidification of powder particles into 3D solids by means of a heat source focused on a specific area [64]. The process currently covers 4 main technology types: SLS, selective laser melting, electron beam melting, and multi-jet fusion [65]. The differences between these technologies are mainly in terms of material applicability, energy delivery methods (including light source type and energy intensity), and other aspects. Despite the different technological paths, all processes follow the principle of “thermally driven layer-by-layer molding—a synergistic action of controlled temperature rise and light source energy to achieve solid construction from powdered materials [66].

Laser-selected sintered technology selectively scans and irradiates the surface of the powder material through a high-energy laser beam in a closed molding chamber, so that the powder particles are fused and bonded according to the preset trajectory, and 3D solids are directly prepared through layer-by-layer molding. After printing, the part is completely covered with powder that has not yet been sintered [67]. The powder compartment needs to cool down for a while before the part can be removed, after which it needs to be further cleaned by sandblasting or using compressed air. Figure 2D and E are the architecture of the SLS printer and the properties of the powder material, respectively [68]. The melting mechanism of powder particles mainly includes 4 types: solid state sintering, chemically induced bonding, liquid phase sintering, and complete melt consolidation. During the printing process, the setting of processing parameters such as temperature, laser beam wavelength, scanning speed, layer height, and particle size will have a substantial impact on the performance of the final molded object. In order to achieve the best use effect of the printed object, the parameters need to be optimized in a targeted manner according to the characteristics of the powder material and the actual application requirements, so that the process parameters can form a good match with the material characteristics and application scenarios [69]. SLS is widely used as a printing material in aerospace, sports equipment, biomedical engineering, and other fields [70,71].

### Fused deposition modeling

FDM, also known as molten material extrusion, is an additive manufacturing technique that relies on thermoplastic filament extrusion [72–74]. After melting into a semi-liquid state, the polymer filament is extruded through a heated nozzle. After being deposited on the build platform, the partially melted filaments solidify, allowing the extruded filaments to layer in turn to form a 3D structure (Fig. 2H and I) [30,56]. FDM is easy to use, simple to operate, and low cost, and is currently widely used in medical, mold design, engineering, and automotive, aviation, and semiconductor devices [75–82].

The print quality of FDM, a typical contact additive manufacturing process, is mainly affected by both machine parameters and process parameters. In terms of machine parameters, bed calibration and nozzle diameter are the most critical considerations [83,84]. Among them, bed calibration is particularly important because the technique requires a constant nozzle-stage spacing to be maintained throughout the printing process. If the calibration deviates, it not only will lead to uneven material deposition and interlayer stress, but also may trigger warping and deformation of the prints and, in severe cases, even cause nozzle collisions. In terms of process parameters, nozzle temperature, bed temperature, extrusion width, and grating angle together determine the final printing effect. Particularly noteworthy is that the choice of nozzle diameter will directly affect the printing efficiency and quality. Although the use of large aperture nozzles can substantially speed up the production time of the part, it will reduce the surface quality at the same time. Therefore, in practice, the best balance between print speed and molding accuracy needs to be achieved according to specific needs [85].

Polymers commonly used in FDM printers are polylactic acid (PLA), acrylonitrile–butadiene–styrene (ABS), polyamide, polypropylene, polyethylene, and polycarbonate [86]. In the FDM-based 3D printing process, PLA has become the most widely used raw material due to its biodegradable and environmentally friendly properties. However, pure PLA polymers have weak mechanical properties and water-soluble defects, which limit their application in FDM printing to a certain extent [87–89]. Therefore, the preparation of PLA composite materials by adding suitable additives has become an effective way to improve the performance of 4D-printed PLA components with the help of FDM technology.

## Magnetically Responsive Materials

Magnetically responsive materials are stimulated by magnetic fields to change their shapes, properties, or functions. For example, magnetic fields and magnetic nanoparticles (MNPs) can be used to establish remote control in miniature receivers made of hydrogels or silicones [90–92]. Compared with other stimulus-responsive materials, magnetic smart materials have great advantages in the following aspects: (a) MNPs are remotely controlled by a magnetic field or magnetic force to deform the material without physical contact; (b) the response speed reaches the order of seconds and the magnetic field has a far range of action, which makes it easy to control the material; (c) parameters such as the magnetic field strength and direction can be precisely regulated by an electromagnetic coil system, which, in turn, enables the material to have precise control; and (d) the magnetic field has a strong penetrating force, and the magnetic field is safe and has a better prospect in the field of biomedicine. In the following, we will introduce the research of several kinds of magnetically responsive materials that have been studied more in recent years.

### Magnetic shape memory alloys

**Fig. 3.**
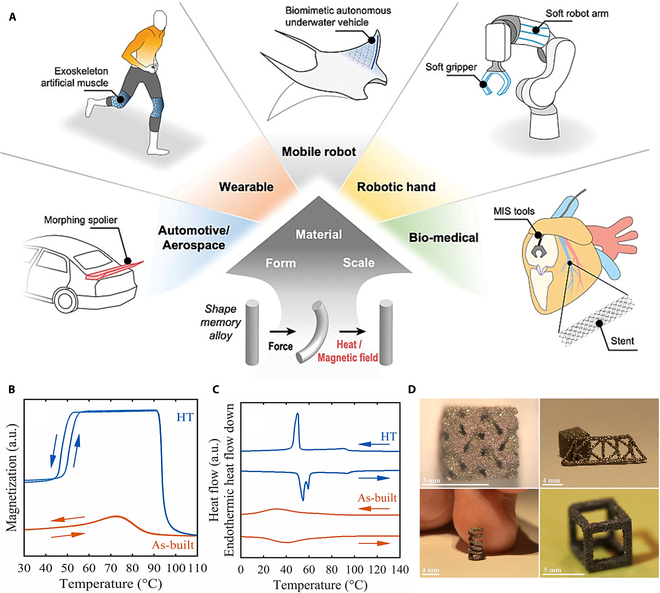
(A) Shape memory alloys in various fields of application [93]. Copyright 2023, Wiley. Properties of a heat-treated sample (HT). (B) Magnetization intensity–temperature curve. (C) Heat flow versus temperature curve [105]. Copyright 2021, Elsevier. (D) Various network structures printed from Ni-Mn-Ga powders [104]. Copyright 2018, Elsevier.

Shape memory alloys are special metal materials that undergo plastic deformation within a certain temperature range and can recover their original macroscopic shape within another temperature range. Compared to other smart materials, shape memory alloys have a higher energy density, more precise control, greater durability, and reprogramming. In many emerging fields such as mobile robots, robotic hands, wearable devices, automotive/aerospace parts, and bio-medical devices, they also show broad application prospects (Fig. 3A) [93].

According to different types of external stimuli, shape memory alloys can be divided into 2 categories: temperature-responsive shape memory alloys (TSMAs) and magnetically responsive shape memory alloys (MSMAs). When an external stress is applied to transform twin martensite into fixed martensite, a deformation cycle begins. When the temperature is higher than the austenite starting temperature (*A*_s_), the martensitic phase (twinning or solidification) begins to transform into the austenite phase, and when the austenite ending temperature (*A*_f_) is reached, the martensite becomes completely austenitic. By cooling SMA to the martensite initiation temperature (*M*_s_), the opposite transformation is achieved. The phase reversal is completed at the martensitic finish temperature (*M*_f_) [93].

The actuation properties of MSMA have a similar material-dependent mechanism to TSMA, and their properties are essentially controlled by the composition and crystallinity of the material. It is worth noting that the operating temperature range of these alloys must be strictly controlled below the martensitic phase transition temperature point (typically <373 K) to prevent thermal perturbations from triggering austenite phase remodeling leading to failure of the actuation function [94,95]. Under the action of magnetic field-induced stress, the crystal structure of the martensitic phase (twin variant) of these materials is rearranged, resulting in large reversible magnetic field-induced strains [11,96]. Compared to heat-activated shape memory alloys such as Ni-Ti, MSMA responds much faster (less than 1 ms compared to a few seconds) [97]. Magnetic shape memory alloys are expected to be used in devices that require high response frequencies, large reversible strains, and high energy efficiency, including micro-actuators, microfluidic pumps, energy conversion devices, and sensors.

In recent years, ternary ferromagnetic Ni-Mn-Ga-Heusler alloys have attracted significant attention. In Ni-Mn-Ga, phase transition occurs from the highly symmetrical L2_1_ austenitic phase to the lower symmetrical martensitic structure. During this phase transition, the arrangement of atoms inside the material undergoes major reorganization. The highly symmetrical L2_1_ phase has a more uniform atomic distribution, whereas the local order is gradually broken during cooling to the martensitic phase, forming an asymmetrical structural feature. This transformation not only affects the macroscopic mechanical properties of the material, such as its strength and toughness, but also substantially alters its thermal and electrical properties. Based on composition and thermal history, 3 different martensitic phases can be distinguished in Ni-Mn-Ga: a modulated pseudo-tetragonal lattice of 10M (c<a exhibiting 3 twin variants), a modulated orthogonal lattice of 14M (c<a exhibiting 6 twin variants), and an unmodulated L10 tetragonal lattice of NM (c>a exhibiting 3 twin variants) [98]. The martensitic phase transition is a diffusion-free primary structural transition, and the atomic arrangement of the martensitic phase is highly similar to that of the austenite; thus, the austenite crystal structure model is usually used to characterize it.

However, monocrystalline Ni-Mn-Ga is difficult to synthesize and costly; polycrystalline Ni-Mn-Ga alloy materials are brittle and difficult to form, making them unsuitable for many engineering applications that currently require relatively high forces; and the magnetic field-induced strain (MFIS) effect of polycrystalline Ni-Mn-Ga bands is almost zero [99,100]. In order to solve the problem of Ni-Mn-Ga materials, 2 solutions have been proposed, one is to mix Ni-Mn-Ga powder with polyvinylidene fluoride (PVDF) polymer to form bulk composites, and the other is to fabricate Ni-Mn-Ga metal foams using ceramic preforms with bimodal pore size distribution [101,102]. It has been found that porous Ni-Mn-Ga has good mechanical properties and large MFIS, which is suitable for engineering applications [103]. Caputo et al. [104] studied the additive manufacturing route of producing functional mesh parts using pre-alloyed magnetic shape memory alloy Ni-Mn-Ga powder by binder jet 3D printing and successfully prepared network porous structures with good mechanical strength (spring-like, 3D hierarchical lattice structure, etc.), proving the possibility of magnetic shape memory alloy 4D printing (Fig. 3D). Laitinen et al. [105] constructed the MSMA Ni_50.5_Mn_27.5_Ga_22.0_ by laser powder bed fusion (L-PBF) using a gas-atomized powder doped with excess Mn, and the samples exhibited a wide austenite-martensitic transition (Fig. 3B) and low saturation magnetization (Fig. 3C). It was shown that stepwise chemical homogenization and atomically ordered heat treatment can substantially enhance their magnetic structural properties, and the analysis suggests that atomic disorder and stress quenching introduced by the L-PBF process are the key factors leading to atypical properties of the initial samples. Future research should focus on the systematic optimization of the L-PBF process to prepare foamy lattice structures with enhanced crystalline weaving.

### Magnetic shape memory hydrogels

**Fig. 4.**
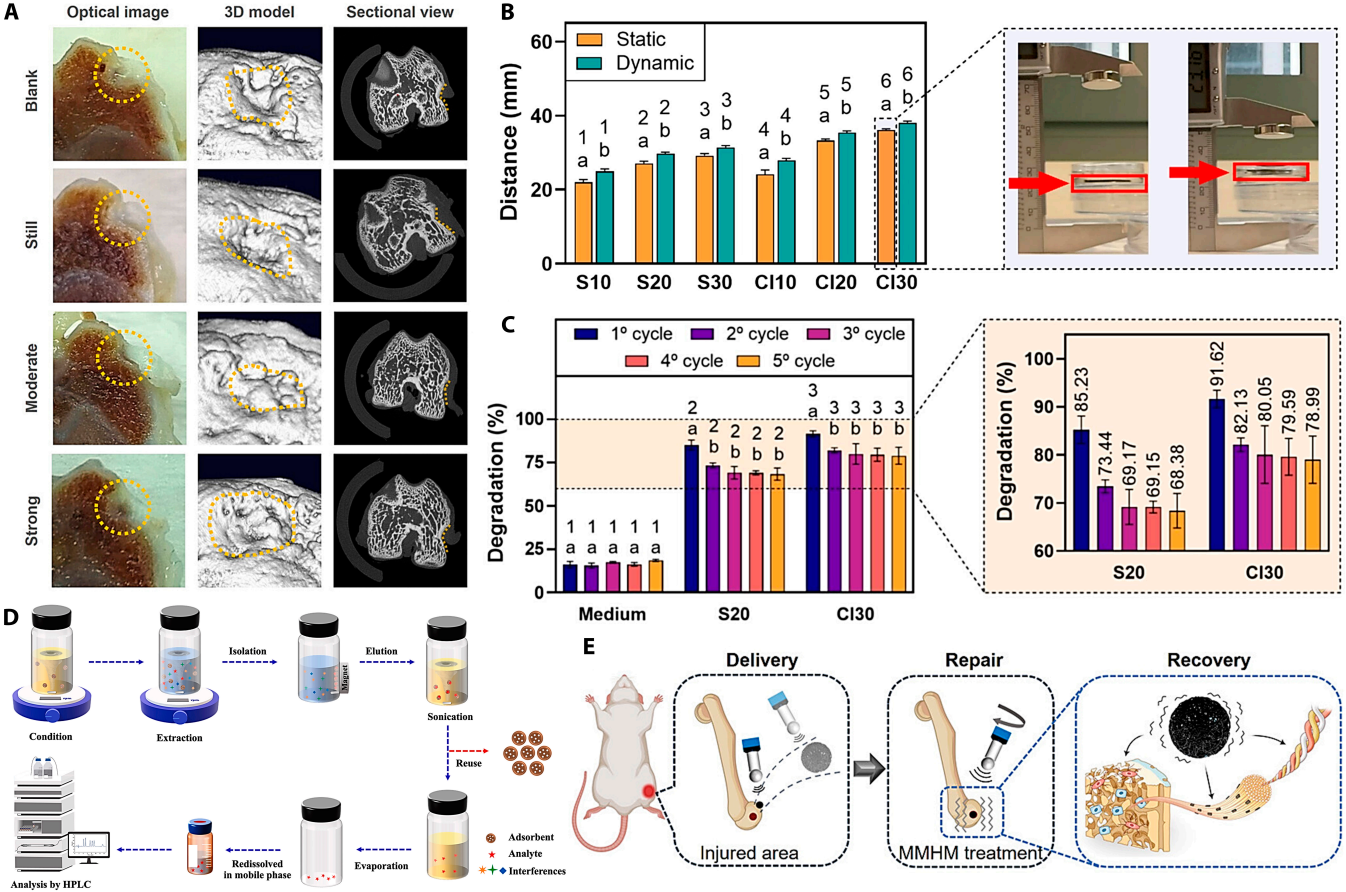
(A) Photographs of the defect area with micro-CT reconstruction (orange dashed line marking the defect) [109]. Copyright 2025, Wiley. (B) Determination of the magnetic attraction distance under static/dynamic conditions and schematic diagram of the device. (C) Degradation rate of S20 and Cl30 hydrogels to XO over 5 cycles [108]. Copyright 2025, Elsevier. (D) Homemade microsphere-dispersed liquid–solid extraction procedure [110]. Copyright 2025, Elsevier. (E) Schematic diagram of bone repair with MMHMs [109]. Copyright 2025, Wiley.

Shape memory hydrogels are a class of smart polymer materials with unique stimulus response properties, and their structure is mainly composed of a permanent cross-linking network and a reversible molecular switch. The permanent cross-linking network determines the initial shape of the material, while the molecular switch is responsible for the fixation and release of the temporary shape [106]. Hydrogels are hydrophilic materials. However, they are insoluble in water because of the cross-linking between polymer chains. Thus, hydrogels can be submerged in water and absorb water until they reach saturation. When the hydrogel dries and is removed from water, the water will leave the polymer matrix and the hydrogel will return to its original shape. This reversible deformation effect allows the use of hydrogels to create self-folding objects [107]. To further expand the functionality of hydrogels, MNPs have been incorporated to create magnetically responsive composites. Viteri et al. [108] synthesized Fe_3_O_4_ nanoparticles in situ within a chitosan-agarose dual-network hydrogel, resulting in a composite material with both catalytic and magnetic properties. The system achieved up to 94% degradation efficiency of organic pollutants under mild conditions (Fig. 4B and C), with excellent magnetic recoverability and reusability. However, its application was primarily limited to environmental remediation, leaving its potential in biological systems unexplored. In order to address this limitation, Shen et al. [109] developed gelatin/hyaluronic acid-based magnetic hydrogel micromotors (MMHMs) using microfluidic techniques (Fig. 4A and E). These micromotors were actuated by an oscillating magnetic field, thereby generating controlled micromotion and enabling bone defect repair without the need for drug loading. This study demonstrated the biocompatibility and biodegradability of magnetic hydrogels in vivo, thus establishing their potential for use in regenerative medicine. As a result of the findings outlined above, Sillapawisut et al. [110] constructed a porous magnetic composite adsorbent by embedding hyper cross-linked polymer-modified graphene oxide and Fe_3_O_4_ nanoparticles into alginate hydrogel beads. The material was applied in dispersive liquid–solid phase extraction for the selective removal of phenylurea herbicides from food samples (Fig. 4D). The composite demonstrated high adsorption capacity, rapid magnetic separation, and consistent performance across multiple cycles, underscoring its practical utility in food safety applications. Collectively, these studies highlight the broad versatility of magnetic hydrogels, which span applications in environmental remediation, biomedical engineering, and food analysis. This multifunctionality is made possible through tailored material design and the strategic integration of functional components. In addition to these emerging uses, shape memory hydrogels have found promising applications in several fields, including electronic devices, sensors, and soft robots [111–114]. Magnetic hydrogels are composed of composites driven by procedures and external magnetic fields by incorporating magnetic particles into precursor gels or solutions. The material demonstrates an ideal pore structure and excellent biocompatibility, while possessing precise magnetic field response properties for remote dynamic modulation in vitro [22,115]. Common magnetic particles include ferrite, superparamagnetic iron nanoparticles, or neodymium particles [116–118].

### Magnetic shape memory polymers and their composites

Shape memory polymers (SMPs) are typically stimulus-responsive smart materials characterized by the ability to transform into a temporary shape when heated above the transition temperature and to fix that shape by applying an external force during cooling. When reheated above the transition temperature, the material can spontaneously return to its initial shape, thus completing a complete shape memory cycle [107,119–121]. These materials combine high stiffness and fast response properties, and are capable of achieving substantial reversible deformation recovery in response to external stimuli [122]. SMP composites are an emerging inelastic structural substance, composed of SMPs and other functional materials, with better mechanical properties that can be restored to their standard shape by changes in the external environment rather than elastic anomalies, and can be used in demanding applications such as medical, origami, sensors, and robotics, as well as aerospace structures such as solar arrays, deployable panels, batteries, booms, self-deploying structures, and reflective antennas [123,124].

**Fig. 5.**
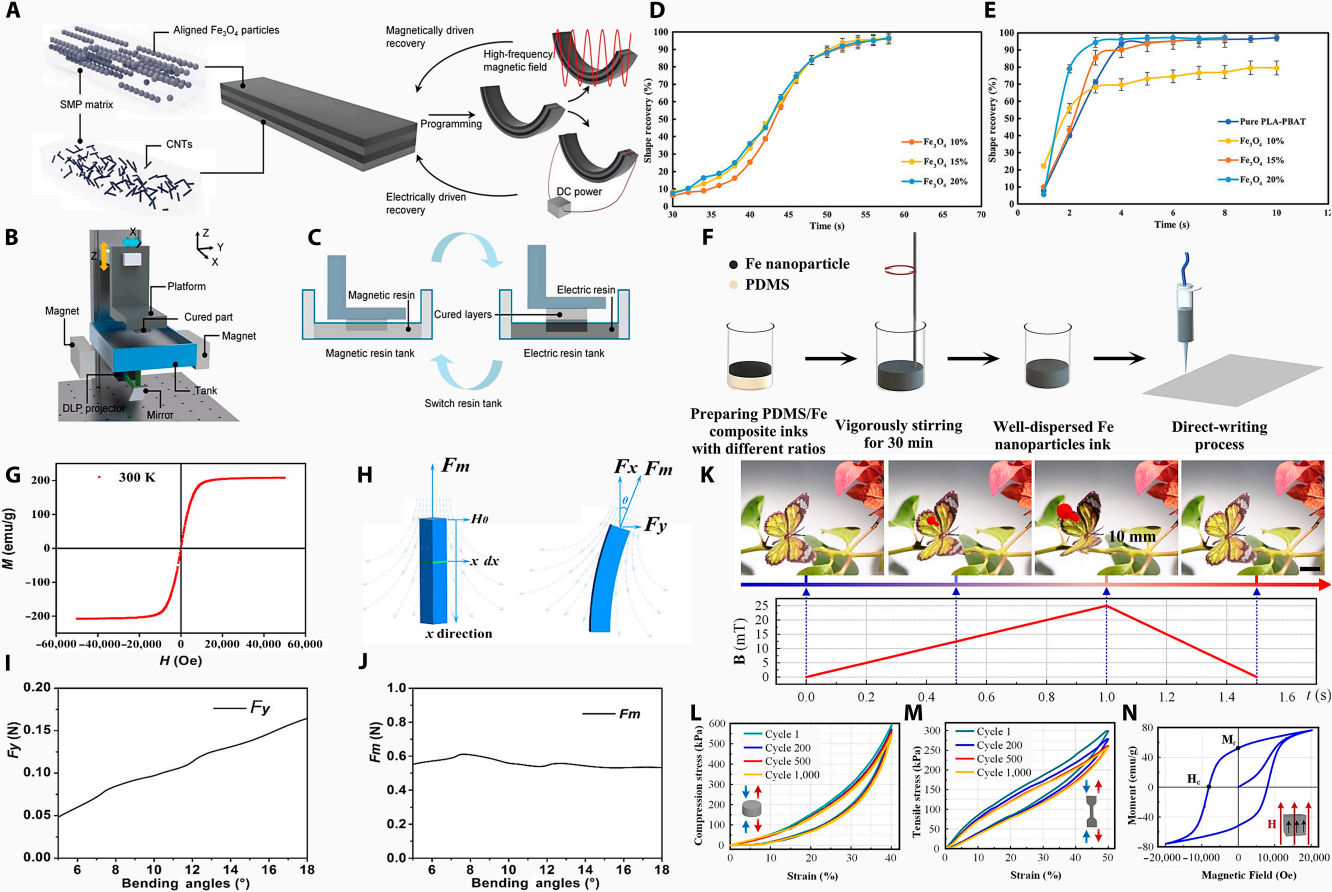
(A) Overall design of ML-EMSMCs. (B) MF-DLP 4D printing equipment and (C) processes [128]. Copyright 2024, Wiley. (D) Hot-water recovery vs. time. (E) Magnetic field recovery vs. time [125]. Copyright 2024, Wiley. (F) Preparation of composite ink and its printing. (G) Relationship between magnetization strength and external magnetic field strength. (H) Schematic of the deformation of a printed square bar under the action of a magnetic field. (I) Horizontal force (*F_y_*) as a function of the bending angle of a square bar. (J) Combined force (*F_m_*) as a function of the bending angle of the square bar [135]. Copyright 2018, American Chemical Society. (K) Wing-vibrating behavior of a bionic butterfly driven by a magnetic field. (L) Stress–strain curve of a cylindrical printed object at 1,000 cycles. (M) Stress–strain curve of a dog-bone-shaped printed object at 1,000 cycles. (N) Hysteresis loops for 4D-printed objects [140]. Copyright 2021, American Chemical Society.

Magnetic SMPs and their composites are mainly composed of an SMP matrix and magnetic fillers, particularly Ni, Fe, and Fe_3_O_4_ nanoparticles, which can change their shape under the stimulation of magnetic fields and maintain a new shape after the stimulation disappears. This material has a shape memory function, which is able to restore its original shape under certain condition. Compared to other advanced materials with stimulus-responsive behavior, magnetic SMPs and their composites have unparalleled properties, such as higher recyclable strain, low density, multiple activation methods, easy processing, and customizable properties, and have received extensive attention in the fields of biomedicine, aerospace, and flexible electronics. Yousefi et al. [125] developed a biodegradable nanocomposite with excellent mechanical properties and shape memory function by melt blending PLA-PBAT with Fe_3_O_4_ nanoparticles. This material can rapidly recover its shape under thermal and magnetic stimulation, exhibiting multi-trigger shape memory capability (Fig. 5D and E). This work provides a new idea for the design of smart materials. However, the addition of high concentrations of Fe_3_O_4_ to the material has been observed to result in nanoparticle agglomeration, which, in turn, leads to a degradation of the material’s properties and an associated reduction in print quality. To address these issues, Mirasadi et al. [126] investigated a PETG-ABS-Fe_3_O_4_ nanocomposite material, which effectively resolved the printing difficulties caused by high nanoparticle content by employing direct particle-based FDM printing technology and regulating the melt flow through a pneumatic system. The material exhibits exceptional 3D and 4D printing capabilities, in addition to its capacity for remote stimulation through a temperature-induced magnetic field. This property signifies its advanced shape memory properties, particularly in response to direct thermal stimulation and magnetic field application. Consequently, it presents a novel alternative for remotely actuated smart materials. In light of the observations derived from the initial 2 materials, Rahmatabadi et al. [127] proceeded to investigate the shape memory effect of PLA-TPU blends. Their findings indicated that the attainment of distinct properties was contingent upon the modulation of the PLA content and the programming temperature. For instance, the PLA70 samples exhibited the most pronounced shape recovery values and stress recovery properties. These findings suggest that the rational design and regulation of smart materials composition and processing can yield shape memory properties, providing a theoretical foundation and practical guidelines for the implementation of 4D printing in biomedical and smart structure domains. In addition, the composite shape memory structure of the spine bone shape was 4D printed, showing the shape unfolding process under a magnetic field. The complex structure of 4D printing has great potential functions in biology and medicine, including but not limited to bone tissue repair. Wu et al. [128] proposed an MF-DLP 4D printing technique to fabricate multilayer electrical/magnetic dual-driven shape memory composites (ML-EMSMCs). Carbon nanotubes (CNTs) and Fe_3_O_4_ particles were embedded as conductive and magnetic fillers in shape memory resins (Fig. 5A to C). The multimaterial printing process is then used to print the conductive and magnetic layers alternately until the entire composite part is completed. Finally, the printed composites have excellent shape memory properties and provide valuable insights for future sensor/actuator applications by demonstrating the selective driving capabilities of printed structures, such as biomimetic petals and fingers.

### Magnetically active soft materials

Magnetically active soft materials (MASMs) are soft elastic composite materials filled with magnetic particles with shape programmability, which are capable of programmable shape transformation and controlled object movement in an unconstrained manner under a magnetic drive [129–131]. In particular, magnetic fields can easily and harmlessly penetrate most materials, providing a safe and effective method of actuation. Magnetic materials can be easily fabricated by simply combining magnetic particles with soft polymers for the unrestricted control of shape changes and movements. Polydimethylsiloxane (PDMS), as a representative silicone elastomer, serves as an ideal matrix material due to its outstanding mechanical properties and chemical stability. When combined with magnetic particles possessing distinct characteristics, such as high-permeability iron (Fe) or high-coercivity neodymium-iron-boron (NdFeB), the composite can meet diverse application requirements for magnetic responsiveness [132–134]. Zhu et al. [135] developed a composite ink system composed of PDMS and iron nanoparticles (Fig. 5F). Among them, PDMS is the flexible matrix component in the composite ink, and the soft magnetic Fe particles can immediately gain or lose their high magnetization strength by turning on or off the external magnetic field owing to their low coercivity and high permeability. The structural evolution of the magnetic response 3D structure printed with PDMS/Fe composite ink has a fast response time under an external magnetic field, and the printed square strip structure can be deformed under the action of a small force, showing the fast response time of the ink under the magnetic field (Fig. 5G to J). In the future, they will have great application prospects in the fields of biomedicine and intelligent manufacturing.

The programmable deformation control of 3D MASM components mainly relies on the spatially encoded arrangement of magnetic particles in the flexible polymer matrix, and the precise shape regulation is achieved by constructing a 3D heterogeneous magnetization field [134,136,137]. In the 4D printing process, the magnetically sensitive particles in the polymer are directed by an external magnetic field to give the pattern magnetic polarity [132,138,139]. However, when a printed magnetic field is introduced, the magnetic particles may be locally clustered because of magnetic attraction, thus affecting the magnetic strength of the printed structure; this situation has a substantial impact on driving efficiency. In the context of uniformly dispersed particles, the external magnetic field has the capacity to establish a coherent magnetic flux path through the synergistic interplay among the particles. This ensures the uniformity of the material’s overall magnetic response. A notable example is the formation of a uniform magnetic dipole array composed of single-domain NdFeB nanoparticles within a gel, which contributes to the maintenance of stable residual flux density and coercive force. This also facilitates the realization of rapid and intricate shape transformation under low magnetic fields. Conversely, particle agglomeration has been shown to induce sudden changes in local permeability, leading to magnetic field distortion and the destruction of uniformity. This, in turn, results in inconsistent response amplitude and speed in different regions. A notable example of this phenomenon is the agglomeration of carbonyl iron powder in magnetorheological elastomers (MREs), which causes abrupt changes in local stiffness and affects the controllability of structural deformation. Additionally, agglomerates generate additional hysteresis loss when the magnetic field undergoes changes, thereby reducing the energy conversion efficiency, particularly under conditions of high-frequency magnetic field drive. The hysteresis of the magnetic response caused by agglomerates leads to a substantial increase in energy consumption. To avoid this problem, Zhang et al. [140] developed a non-magnetized 3D injection printing ink containing NdFeB hard magnetic particles, which can effectively avoid the local agglomeration of NdFeB particles under the action of magnetic fields. By combining traditional 3D injection printing with origami magnetization technology, MASM objects with 3D pattern magnetization profiles can be easily manufactured. The printed structure quickly returned to its initial shape after the compression and tensile cycling tests, exhibited good elastic properties, and exhibited excellent ferromagnetic properties (Fig. 5L to N). By simulating the flapping process of a bionic butterfly over a certain period, reversible magneto-induced deformation was demonstrated (Fig. 5K). Magnetic soft materials can be rapidly deformed under the remote driving of magnetic fields, showing potential application prospects in flexible electronics, biomedicine, and other fields. Kim et al. [134] reported tether-less 3D-printed soft materials that rapidly transition by magnetic fields. A magnetic field was applied to the dispensing nozzle when the DIW printed elastomer composites containing ferromagnetic particles. The ferromagnetic particles redirected along the applied magnetic field give the printed filament pattern magnetic polarity, allowing programming of ferromagnetic domains in complex 3D-printed soft materials. The ferromagnetic domains programmed in the printed soft material enable fast transitions between complex 3D shapes using a direct magnetic drive. Wajahat et al. [141] developed a novel 4D printing strategy to successfully prepare a variety of MSM 3D structures of different shapes by using DIW printing techniques to construct the structure of the material using a DIW printing technique using an ink composed of NdFeB and styrene-isoprene block copolymer (SIS) at room temperature, and then programming and reconstructing the magnetization curves of the printed structures using an origami magnetization method to the desired shape. The results indicated that this strategy has great potential for the fabrication of multifunctional, deformed, and reprogrammable magnetically active devices for advanced soft actuator applications.

## Applications of 4D Printing Magnetically Responsive Materials

In recent years, 4D printing technology has attracted a lot of attention in many research fields, including smart devices, metamaterials, biomedical engineering, and other fields, showing a wide range of potential applications [142–145].

In the field of intelligent devices, such as soft robots, Wehner et al. prepared the robot with the help of combinatorial manufacturing techniques. First, they built the robot’s body using a molding process. Then, they used soft lithography to build microfluidic logic. Finally, a network of pneumatic actuators, an on-board fuel depot, and a catalytic reaction chamber were fabricated through multimaterial embedded 3D printing. After these steps, an autonomous robot composed entirely of soft bodies is prepared [143]. In the field of functional devices, 4D printing has great potential for use in sensing, energy harvesting, and storage [146–156]. For example, 4D-printed actuators are perfect for creating stimulus-responsive soft robots that can excel in energy storage and harvesting [157]. In biomedical engineering, customized sutures, therapeutic devices for ischemic stroke, vascular repair devices, and thermo clamps can be realized with 4D printing, leading to more innovative solutions in the medical field [158–161].

4D printing technology based on magnetically responsive smart materials is becoming a research hotspot in the field of smart materials, which realizes precise deformation control of complex structures through non-contact magnetic field regulation. The technology utilizes the synergistic control of magnetic field strength and frequency to realize unconstrained and high-precision remote operation, showing unique advantages in minimally invasive medical treatment, special robotics (extreme environment operation), and other fields. Compared with traditional driving methods, magnetically responsive 4D printing has substantial safety advantages, especially for in vivo medical scenarios. However, this technology still faces challenges such as material properties and imperfect multi-field coupling theory [22]. Therefore, this section will systematically describe the innovative applications of magneto-responsive smart material 4D printing in biomedical, robotics, and other fields.

### Biomedical tissue engineering

As a cutting-edge manufacturing technology, 3D printing has revolutionized the biomedical field owing to its breakthrough spatial resolution and unprecedented design freedom. It enables the precise customization of a range of biomedical devices, such as scaffolds, implants, prostheses, and surgical tools, to meet individual needs in clinical practice [162–165]. 3D bioprinting is a major breakthrough and advancement in traditional 3D printing technology. At its core, it aims to encapsulate living cells directly into the architecture of tissues such as the liver, heart, and blood vessels in an attempt to create biologically active structures by mimicking the way the human body builds its natural tissues. This technological leap is expected to reshape the future direction of biomedical engineering by providing potential solutions for organ transplantation, tissue repair, and many other challenges. The application of 3D printing technology in the biomedical field enables the customization and personalization of tissues, pharmaceuticals, and medical devices. However, a significant number of biomedical applications necessitate dynamic shape modifications, a feat that conventional 3D printing techniques are incapable of achieving. Although 3D printing technology can use biological materials and living cells to build heterogeneous tissues and organs, static 3D printing structures are difficult to match the dynamic characteristics of natural tissues, nor can they meet the functional requirements of practical applications. Because biological tissues have dynamic properties and are in a continuous transformation process, more complex manufacturing techniques are required. In response to this demand, 4D printing technology came into being, expanding the traditional 3D space to a fourth dimension that includes the time dimension. 4D printing uses shape memory smart materials, which are able to respond to external stimuli by undergoing reversible deformations under predetermined conditions and, thus, are uniquely suited for a wide range of complex applications. In particular, in the field of biomedical tissue engineering, 4D printing is capable of applying forces to biological tissues and cells to enable precise control and therapeutic manipulation where biocompatibility is required [166].

The biocompatibility of materials is a primary consideration in biomedical applications, especially where in vivo use is involved. This makes magnetically responsive smart materials a highly interesting research direction, as they are capable not only of remote manipulation through magnetic fields in non-contact situations but also of precise adjustment and deformation of target objects through magnetic stimulation. The advantage of magnetically responsive materials is that they can be rapidly and reproducibly actuated in in vivo or in vitro environments by changing the external magnetic field without the limitations of traditional electrical or mechanical control methods. Therefore, the potential of magnetic materials in the biomedical field, particularly in applications such as endoscopy, stent therapy, robotic surgery, and cellular regulation, shows an extremely promising future. The multifunctionality of magnetically responsive materials can be further enhanced by compositing them with other smart materials (e.g., SMPs and liquid metal hydrogels), which can drive the progress of precision and personalization of smart medical devices. Table 2 presents part of the research status of magnetically responsive materials in biomedical tissue engineering.

**Table 2.** 4D printing of magnetically responsive materials for medical tissue engineering applications

Materials	Printing technology	Response mechanism	Limitations	Applications	Reference
Magnetic hydrogel	DLP	Oriented arrangement of magnetic nanoparticles	Complex process	Heterogeneous sports brackets	[167]
Magnetic hydrogel	DIW	Gradient distribution of magnetic nanoparticles	Low accuracy and weak stability.	Bionic “hand-waving” structures	[168]
Magnetic polymer composites	DIW	Thermal effects in Fe_3_O_4_ nanoparticles	Deformation temperature may damage cells	Endovascular stents	[169]
Magnetic polymer composites	FDM	Thermal effects in Fe_3_O_4_ nanoparticles	High-temperature deformation may damage cells	Bionic tracheal stent	[172]
Magnetic polymer composites	DIW	Near-infrared/alternating magnetic field triggered shape recovery	Degradation rate not matched to osteogenesis rate	Bone regeneration scaffold	[173]
Magnetic Polymer composites	/	Heat generation in Fe_3_O_4_ nanoparticles	Degradation rate not matched to osteogenesis rate	Composite stents (tumor therapy)	[170]
Mineralized composites	Microfluidic 3D printing	Heating promotes osteogenic differentiation	Complex and costly process	Bionic mineralized bone scaffold	[174]
Magnetic hydrogel	DIW	External static magnetic field activates Fe_3_O_4_@CS	Mechanism remains unclear	Ear cartilage scaffolds	[175]
Magnetic polymer composites	DIW	Near-infrared/alternating magnetic field triggered shape recovery	Long wet irradiation time	Bone repair and anti-tumor	[171]

By compounding MNPs with biocompatible polymers, the researchers have developed a series of smart scaffolds that can be remotely manipulated to optimize the functionality of the entire process, from minimally invasive implantation to tissue regeneration.

**Fig. 6.**
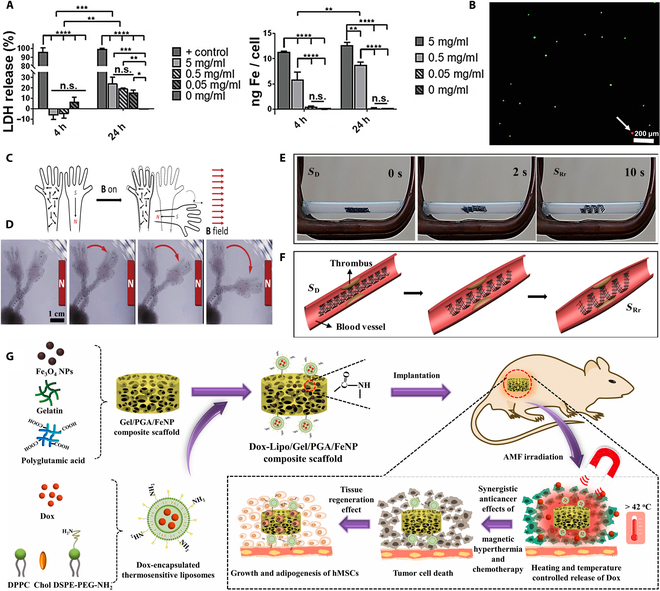
(A) Lactate dehydrogenase (LDH) release (toxicity) and Fe uptake of hMSCs after 4- and 24-h exposure to different concentrations of MNPs. (B) Live (green)/dead (red) staining of humandermal fibroblast cultured on composites with 5 wt.% nanoparticle loadings. (C) Schematic diagram of the principle of stent deformation driven by magnetic field. (D) Oriented magnetic nanoparticles can drive nanocomposites to move in different directions [167]. Copyright 2022, Wiley. (E) Schematic of shape recovery of a printed stent under an alternating magnetic field. (F) Potential application of stents in blood vessels [169]. Copyright 2017, American Chemical Society. (G) Preparation of composite scaffolds and schematic illustration of their application to magneto-thermal therapy and controlled chemotherapy [170]. Copyright 2024, Elsevier.

At the material design level, the synergy between magnetic responsiveness and bio-adaptability has become a key breakthrough. For example, Tobias Kuhnt et al. constructed a rigid structure of PCTAc and a flexible hydrogel of PEGDA with heterogeneous actuation capability by aligning anisotropic iron oxide MNPs by magnetic field orientation and combining it with DLP printing technology. The orientation of the rigid structure can show characteristic attraction or repulsion depending on the direction of the magnetic field, and the flexible hydrogel enables complex deformation to prepare scaffolds with heterogeneous and complex motions (Fig. 6C and D). In terms of biocompatibility, they exposed human mesenchymal stem cells (hMSCs) to different concentrations of MNPs and cultured them for 4 and 24 h, and found that the cytotoxicity of the MNPs at the low-concentration dose was negligible, which fulfills the actual scaffolding requirements and demonstrates excellent biocompatibility (Fig. 6A and B) [167]. Similarly, Simińska-Stanny et al. [168] achieved multimodal motions such as rolling and bending by multimaterial printing using patterned magnetic hydrogels, providing new ideas for minimally invasive surgical instruments. Wei et al. [169] demonstrated shape-changing structures with active shape changes through ultraviolet (UV) cross-linked poly(lactic acid)/Fe_3_O_4_ composite inks for thermally and remotely driven SMP, utilizing the hysteresis effect to remotely heat Fe_3_O_4_ nanoparticles under an alternating magnetic field. The printed 3D scaffolds provide magnetically guided and remotely actuated behavior (Fig. 6E) and can be used as endovascular scaffolds (Fig. 6F) with potential biomedical applications.

**Fig. 7.**
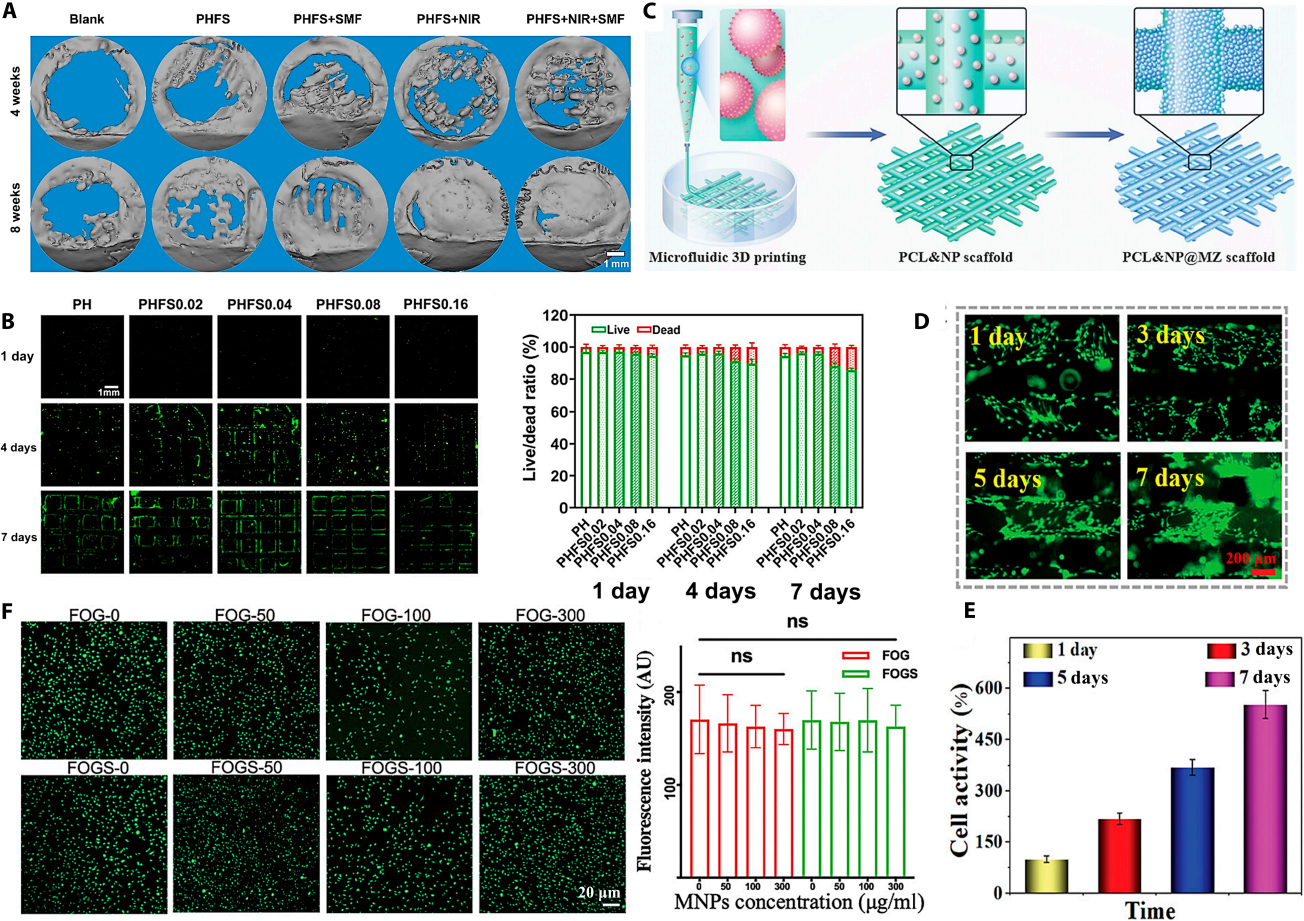
(A) Micro-CT images of new bone regeneration from in vivo scaffolds. (B) Cell viability testing of samples [173]. Copyright 2024, Elsevier. (C) Fabrication of PCL&NP@MZ scaffold from microfluidic 3D printing. Hydrophilicity and cell activity test of PCL@MZ scaffold. (D) The cell growth behavior during 1 to 7 days’ culture on the PCL@MZ scaffold. (E) The cell activity on the PCL@MZ scaffold from day 1 to day 7 [174]. Copyright 2025, Wiley. (F) Cell survival under culture conditions with different concentrations of MNPs [175]. Copyright 2025, Wiley.

In clinical application scenarios, the combination of magneto-thermal synergistic effect and bionic structure substantially improves the therapeutic effect. In tumor therapy, Sun et al. innovatively hybridized magnetic Fe_3_O_4_ nanoparticles, heat-sensitive liposomes loaded with Adriamycin (Dox), and biodegradable polymers to successfully prepare a smart composite scaffold. The scaffold adopts the dual-function design of “simultaneous activation of magneto-thermal therapy and chemotherapy” and “in situ tissue regeneration”, which can not only realize the effect of magneto-thermal therapy by raising the temperature under the action of an applied alternating magnetic field, but also trigger the release of Adriamycin to play the chemotherapeutic effect (Fig. 6G). This achievement provides a brand-new solution for the postoperative treatment of solid tumors such as breast cancer [170]. In addition to this, Wang et al. constructed a hierarchical porous shape memory scaffold consisting of hydroxyapatite, silica, poly(D,L-propylidene-co-tri-methylene carbonate), and Fe_3_O_4_ (HSP-Fe_3_O_4_) by 4D printing, which can provide effective photothermal conversion for photothermal therapy under near-infrared laser irradiation as well as magnetothermal therapy at an alternating magnetic field, providing positive insights into the prevention of tumor recurrence and promote bone regeneration after osteosarcoma surgery, providing positive insights [171]. In the field of trachea repair, inspired by glass sponges, Zhao et al. [172] 4D-printed PLA/Fe_3_O_4_ bionic trachea scaffolds are both torque-resistant and magneto-thermally responsive, with in vivo shape restoration in 35 s, and their helical ridge structure further optimizes the mechanical support performance. In terms of bone repair, the Fe_3_O_4_@SiO_2_ composite bone scaffold developed by Zhang et al. can precisely match irregular bone defects through the dual-response mechanism of near-infrared light/static magnetic field and activate the phosphatidylinositol 3-kinase/protein kinase B (PI3K/AKT) pathway to promote bone regeneration (Fig. 7A), resulting in a substantial increase in osteogenic efficiency. The excellent biocompatibility of the scaffolds with 4 wt.% Fe_3_O_4_ was also demonstrated by evaluating the concentration of nanoparticles on the cells, and cell viability on the scaffolds using the Live/Dead staining kit (Fig. 7B) [173].

In addition, material functionalization modification further expands the therapeutic dimension. Shang’s team introduced MgSiO_3_@Fe_3_O_4_ nanoparticles into polycaprolactone (PCL) scaffolds, which, combined with the bionic mineralized (MZ) layer technology (Fig. 7C), not only enhanced cell adhesion (Fig. 7D and E), but also synergistically promoted osteogenic differentiation through the magneto-thermal effect, which provides a new way to provide bone repair. “Material–structure–function” integrated solution for bone repair [174]. Zhang et al. proposed a “multifunctional composite system” as a scaffold suitable for ear cartilage tissue engineering. By combining in vitro cultivated chondrocytes with magnetic hydrogel, a new magnetic hydrogel scaffold was printed, which addresses 3 key aspects of cartilage tissue regeneration: chondrocyte proliferation, inhibition of graft-associated inflammation, and external regulation of cartilage tissue. To evaluate the biocompatibility of Fe_3_O_4_, chondrocytes were cultured by placing hydrogel precursors containing different concentration gradients of Fe_3_O_4_ in petri dish species, which did not show substantial cytotoxicity after 48 h, and are expected to provide new opportunities for the application of ear cartilage tissue engineering (Fig. 7F) [175].

Through the triple advantages of magnetic field driving, structure bionic design, and bioactivity regulation, 4D-printed magnetic smart materials promote the development of tissue engineering in the direction of minimal invasiveness, personalization, and functionalization. In the future, by optimizing the suitability of material components and printing processes, such materials are expected to achieve precision medical applications in more complex pathological environments.

### Robotics area

The employment of 4D-printed magnetically responsive materials in the domain of bionic robots has demonstrated their remarkable capacity to emulate biological movement patterns and augment the adaptability of robots. These robots are capable of navigating complex and dynamic environments with ease, achieving operations that are both efficient and precise by emulating the movement and behavioral patterns of organisms found in nature.

#### Bionic robot

**Fig. 8.**
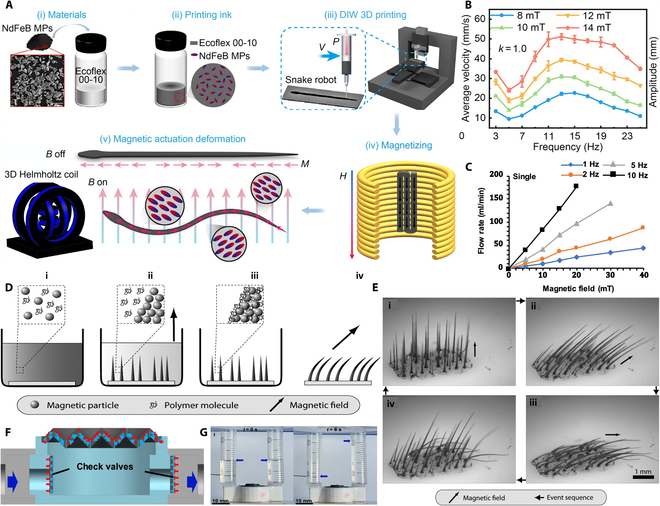
(A) Snake-robot fabrication schematic. (B) Swimming speed rises with field strength and peaks at medium frequency [176]. Copyright 2025, Springer Nature. (C) Ideal in-/out-flow curves of a diaphragm pump under varied conditions [178]. Copyright 2023, Springer Nature. (D) Dilute magnetic–levitation assembly. (i) Mixed, (ii) field-induced phase separation, (iii) post-solvent, and (iv) functional at any field angle. (E) 450 mT/20 T m^−1^ gradient yields bent magnetic cilium, erects at 90°, bends at 45° or 0°, recovers after field-off, and re-erects on re-application [177]. Copyright 2010, American Chemical Society. (F) Single-diaphragm pump layout. (G) Pump delivers unidirectional flow at 1 Hz, ±40 mT [178]. Copyright 2023, Springer Nature.

Among the many research results, the preparation technology of 4D-printed bionic snake-like soft robot designed by Prof. Liu Tianx’s team at Jiangnan University is particularly outstanding [176]. The team prepared a gel-like magnetically responsive smart ink with shear-thinning and shear-thickening properties by uniformly mixing NdFeB magnetic particles with uncured polymer (Fig. 8A). Using a self-developed open-source DIW 3D printing system, the ink was deposited layer by layer along a predefined path, and subsequently cured and molded at room temperature. The critical magnetization step was performed by a capacitive pulse magnetization device, which enabled the robot to achieve saturation magnetization. Under the precise control of the dynamic magnetic field generated by the custom 3D Helmholtz coil system, the snake robot successfully achieved complex dynamic behaviors including straight-line swimming, turning, circling, and clustered cooperative motions. At a magnetic field strength of 14 mT and a frequency of 13 Hz, the robot achieved a straight-line swimming speed of 51.159 mm/s (1.705 BL/s) (Fig. 8B). The experimental results further show that the robot maintains its initial deformation capability after more than 30,000 deformation cycles, demonstrating excellent durability.

In terms of the complexity of the preparation method, the 4D printing technique of Prof. Tianxi Liu’s team, while capable of realizing complex structures and functions, requires higher equipment and processes. In contrast, the template-free magnetic assembly method used by the research team led by Jaakko V. I. Timonen and Robin H. A. Ras is simpler (Fig. 8D) [177]. They mixed micron-sized ferromagnetic particles with elastomeric polymers and dried them in an external magnetic field to form magnetic cilia that are mechanically stable even in the absence of an external magnetic field and a solvent, with lengths of up to millimeters, aspect ratios of more than 100, and adjustability by magnetic field gradient and particle size. Experiments have shown that these magnetic cilia are capable of reversible bending in response to a magnetic field, generating a bending drive force sufficient for translation of macroscopic non-magnetic objects and mixing of highly viscous liquids (Fig. 8E). The mechanical properties of the magnetic cilia can be easily tuned by varying the polymer type, showing promise for a wide range of applications, such as microfluidics. This facile preparation method lowers the technological threshold and opens up the possibility of a wide range of applications for magnetically driven micro- and nanorobotics.

Further analyzed, the template-less approach of Timonen and Ras’s team, while simplifying the preparation process, has limitations in terms of structural complexity and functional diversity. In contrast, the monolithic molded folded diaphragm proposed by a joint team from Xi’an Jiao tong University, City University of Hong Kong, and the Max Planck Institute for Intelligent Systems, Germany, compensates for this deficiency to a certain extent (Fig. 8F) [178]. They used NdFeB magnetic particles mixed with silicone elastomer at a mass ratio of 1:1, and through specific mold casting, removal of air bubbles, and pulsed-field magnetization treatment, the diaphragm achieved a structure with radial magnetization under a uniform magnetic field of 40 mT, demonstrating 3D, bidirectional large deformations as well as the ability to substantially change the internal volume (Fig. 8G). Based on the folded diaphragm, the research team designed 3 application systems: a diaphragm pump, an earthworm-like crawling robot, and a squid-like swimming robot. Among them, the single-chamber diaphragm pump can achieve a maximum flow rate of 178.1 ± 3.5 ml/min under a ±40-mT magnetic field, and the dual-chamber pump can achieve a maximum flow rate of 61,394 ± 748 ml/min/kg under a magnetic field of −15 to 15 mT, 5 Hz, demonstrating excellent lightweight and high-power output performance (Fig. 8C). The earthworm-like robot can crawl at a speed of 30 mm/s in a 150- to 40-mm pipe under a −40- to 40-mT, 2-Hz magnetic field. Folded diaphragms and their application systems have promising applications in soft-body robotics due to their simple preparation method and excellent magnetic actuation performance. This approach substantially increases the functional diversity and complexity while maintaining a relatively simple preparation process.

In conclusion, the 2-step molding process used by Guo Zhan Lum’s team reaches new heights of structural precision and functional integration. They mixed NdFeB particles with silicone rubber to form the active component, and mixed aluminum powder with silicone rubber to form the passive component. The negative mold is processed by a computer numerical control machine tool and poured into the passive component for curing, and then the non-uniform width area is cut by laser and poured into the active component for replacement, forming a composite structure with uniform thickness. Finally, a laser cutting jig is used to bend/fold the beams during magnetization, giving the directional profile of the magnetic moment. The jellyfish robot is able to achieve propulsion at the oil–water interface through oscillating tentacles with an average speed of 1.8 mm/s and can be steered by changing the direction and gradient of the magnetic field, demonstrating good directional control. This 2-step molding process not only achieves precise structural control, but also enhances the functional integration of the robot through the combination of active and passive components.

To summarize, these research teams have successfully developed a variety of magnetically driven bionic robots through their distinctive preparation techniques and methods. From the 4D-printed snake robot by Prof. Tianxi Liu’s team, to the template-free magnetic assembly of artificial cilium by Timonen and Ras’s team, to the folded diaphragm and its application system by the joint team, and the 2-step molded jellyfish robot by Lum’s team, these results not only have their own innovations in preparation process, but also show great potentials in function realization and application expansion. The successful development of these magnetically driven bionic robots provides a solid technological foundation and broad imagination for future applications in biomedicine, microfluidics, environmental monitoring, and many other fields. From complex 4D printing technology to easy template-less assembly to precise 2-step molding process, these researches not only promote the development of magnetically driven soft robotics, but also provide diversified solutions for different application scenarios.

#### Medical microrobots

The use of 4D-printed, magnetically responsive materials in medical microrobotics has revolutionized medical technology. These microrobots can perform tasks in the complex physiological environment of the human body and precisely localize and treat diseased areas through precise control of external magnetic fields. In today’s rapidly developing field of medical technology, medical microrobots are emerging as cutting-edge innovations. The clever application of 4D-printed, magnetically responsive materials is undoubtedly an influential breakthrough in this field, providing a powerful impetus for upgrading medical technology as a whole. In the complex and sophisticated internal physiological environment of the human body, medical microrobots perform critical tasks. With precise control of an external magnetic field, they can act like precision-guided missiles, striking directly at diseased areas and enabling precise positioning and treatment of diseases. This greatly enhances the relevance and effectiveness of medical interventions.

**Fig. 9.**
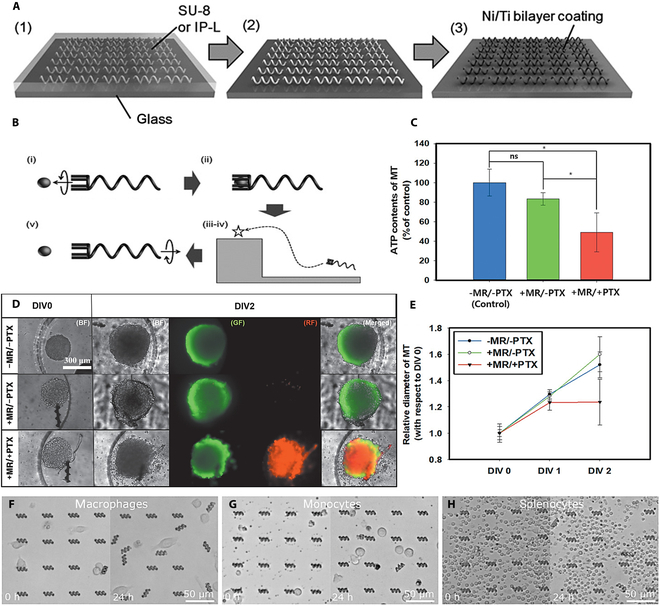
(A) Process flow for the preparation of spiral-shaped swimming microrobots. (B) Schematic diagram of transportation using a spiral-shaped micromachine with a micromount [179]. Copyright 2012, Wiley. (C) ATP contents of the HCT116 MTs. Results are presented as means ± standard deviation (*p < 0.05; ns, *p* > 0.05; *n* = 3). MR, microrobot. Scale bar: 300 μm. (D) Bright-field and fluorescence images of the HCT116 MTs under perfusion conditions in a gravity flow system at DIV0 and 2. (E) Relative diameter of the MT at DIV2 [180]. Copyright 2020, Wiley. (F) Macrophages, (G) monocytes, and (H) splenocytes interacting with arrays of zwitterionic (S30) stealth microrobots for 24 h [181]. Copyright 2020, Wiley.

The spiral-shaped microrobot developed by Tottori et al. [179] excels in this field (Fig. 9A). The innovative design realizes flexible control of swimming movements and cargo transportation process in an alternating magnetic field through the synergistic working mechanism of a miniature scaffold and a spiral structure. The entire transportation process can be regarded as a carefully choreographed dance, going through 4 key stages in sequence: approaching the target area, loading the cargo accurately, transporting the cargo stably, and releasing the cargo in a timely manner as needed (Fig. 9B). These phases are closely intertwined to ensure that the therapeutic substance is delivered to the exact location of the lesion.

However, although the helical microrobots pioneered by Tottori et al. have achieved milestones in magnetically driven navigation and cargo delivery, their carrier materials are still based on light-curing resins, which are inevitably recognized and cleared by the immune system during long-term in vivo cycling, thus greatly limiting the therapeutic time window. To overcome this bottleneck of immune interception, Lee et al. [180] introduced an amphiphilic hydrogel system for the first time in a 4D printing framework and constructed an “invisible” helical microrobot in a single step through 2-photon polymerization. The material programmatically embedded superparamagnetic iron oxide nanoparticles in the cross-linked network, which reproduced the helical swimming pattern of the Tottori robot under an alternating magnetic field. More importantly, the amphiphilic surface was hardly phagocytosed in macrophage co-cultures for more than 90 h (phagocytosis rate <2%), whereas the conventional group was close to 100% (Fig. 9C to E). With the “time–response” dimension of 4D printing, the research team also encapsulated biologically active molecules such as Adriamycin and fluorescent proteins inside the robot in the same printing step, and realized on-demand release of near-infrared through the photocleavage linkage arm, completing the transition from invisibility to precision. This has completed the closed-loop upgrade from invisibility to precise treatment.

Building on this foundation, Cabanach et al. [181] have further integrated the magnetic response concept of 4D printing with the “mechanical anchoring” strategy to develop a needle-like microrobot. The structure capitalizes on the programmable shape memory effect inherent in 4D printing, utilizing this effect to approach tumor microtissues with a flexible movement guided by a magnetic field. Subsequently, the structure triggers local hardening, which serves to “pierce” the lesion as a microneedle and anchor it for sustained drug release (Fig. 9F to H). In comparison to Cabanach’s “total invisibility” approach, Choi’s design emphasizes the integration of “reach–anchor–treat”. The microneedle material is a biodegradable magnetic alloy/polymer composite ink, which gradually degrades after the task is completed, thus avoiding long-term retention. In this manner, 4D printing confers upon microrobotics the spatial and temporal control of “when–where–how” to act. Furthermore, it solves the 3 major problems of immune escape, precise localization, and controlled degradation through the synergistic design of materials, structures, and functions.

It is evident that 4D printing technology has facilitated significant advancements in the field of microrobotics, enabling the realization of complex functionalities that were previously unattainable. For instance, the integration of magnetically driven helical structures, amphiphilic ion cloaking platforms, and needle-like anchoring systems has enabled microrobots to achieve the 3-stage transition of “able to move, able to hide, and able to anchor”. This remarkable development underscores the potential of 4D printing technology in shaping the future of microrobotic applications. The core of the project lies in the deep integration of the spatial dimension, the temporal dimension, and the material dimension (invisible hydrogel and degradable magnetic composite ink). In the future, with the further expansion of 4D printing ink to multi-stimulus response, multi-scale gradient, and biodegradation, the microrobot is expected to realize the ultimate vision of “long flight time, zero immunity, and accurate treatment” in real physiological environments, which will truly open up the 4.0 era of non-invasive or minimally invasive medical treatment. Advances in related technologies have been continuous and marked by substantial improvement. It is anticipated that the development of medical microrobotics will have a considerable impact on biomedical technology, with the potential to substantial enhance human health and promote the growth of the medical industry. The future of medical technology is expected to be characterized by increased accuracy, efficiency, and intelligence. As related technologies continue to progress and improve, medical microrobots will undoubtedly advance biomedical technology to new heights, creating more miracles for human health and promoting the development of the medical industry toward greater accuracy, efficiency, and intelligence.

### Smart structures and devices

By introducing the “time dimension” into additive manufacturing, 4D printing enables structures to dynamically evolve under external stimuli, while magnetically responsive materials become the core carrier of smart structures and devices by virtue of their non-contact manipulation, millisecond response, and deep tissue penetration. Driven by magnetic field, 4D-printed magnetically controlled smart structures can break through the limitations of traditional static devices to realize adaptive deformation, variable stiffness regulation, and self-awareness function integration, bringing breakthroughs in the fields of flexible sensors and functional devices [182–185].

#### Flexible sensors

Flexible sensors are flexible, stretchable, and bendable sensors that can adapt to different shapes and surfaces, and can be used to measure a wide range of physical quantities such as strain, pressure, temperature, humidity, and so on [186,187]. Adding magnetically responsive materials to flexible sensors can make the sensors responsive to magnetic fields, thus enabling detection of magnetic fields, magnetic field-driven deformation or motion, etc. The combination of magnetically responsive materials and 4D printing technology makes it possible to integrate multiple sensing functions into a single flexible structure and give it smart response properties, which presents great potential for applications in wearable devices, environmental monitoring, and other fields.

**Fig. 10.**
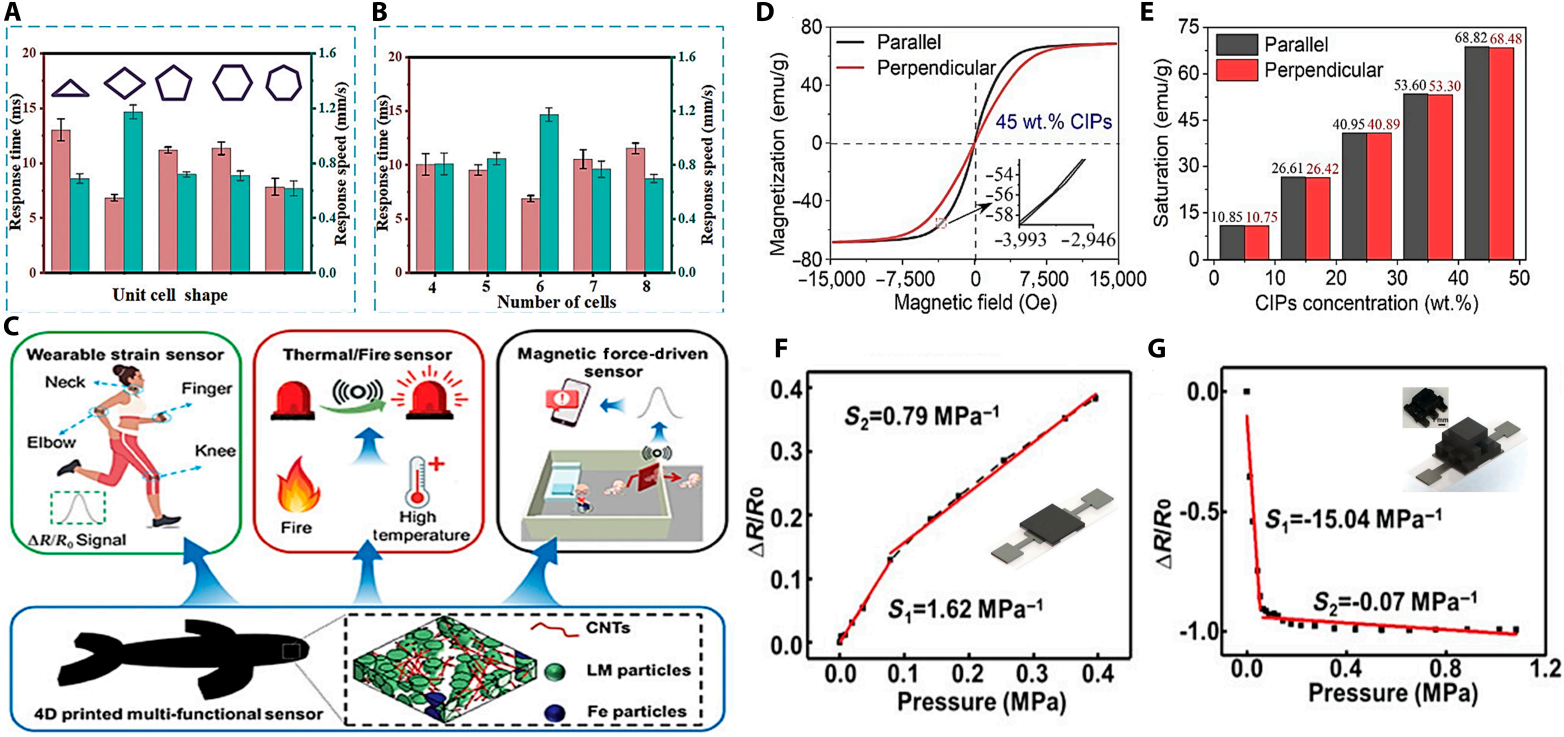
The deformation response time (orange) and average deformation response speed (green) of the EMA for 4D-printed scaffolds’ different (A) cell shape and (B) cell number [3]. Copyright 2024, Wiley. (C) Multi-functionality of 4D-printed sensors [188]. Copyright 2025, Wiley. (D) Hysteresis lines of samples. (E) Saturation magnetization of samples at different CIP concentrations [189]. Copyright 2023, Taylor & Francis. (F) Relative resistance versus pressure for a stacked structure sensing cell. (G) Relative resistance versus pressure for a solid structure sensing cell [191]. Copyright 2022, Wiley.

In recent years, considerable attention has been focused on smart materials as a pivotal factor influencing the performance of flexible sensors. Zhou et al. developed a composite resin by mixing CNTs, liquid molding agent, and micron-sized iron powder with Ecoflex 00-10. The uncured resin was shaped into the desired form by DIW and subsequently cured through the application of heat. It is evident that, with effective management of the composition, a distinctive equilibrium of high sensitivity, stability, and versatility can be accomplished. This, in turn, allows sensors to fully realize their potential in multifunctional sensing devices. The incorporation of iron particles gives the material magnetic response properties, thus integrating strain, temperature, magnetic field detection, and other sensing functions within a single highly flexible structure. When strain sensing and magnetic sensing functions are combined in a thermally stabilized elastomer material, the material can demonstrate excellent versatility and environmental adaptability in diverse application scenarios (Fig. 10C) [188]. Wu et al. successfully developed a soft magnetic resin using a 4D printer to achieve high flexibility and stretchability. Cyclic tensile tests showed that the prepared flexible anisotropic soft-magnetic composite had excellent mechanical properties and durability, as well as good magnetic properties (Fig. 10D and E) and robustness, providing insights into the external field-assisted additive manufacturing of anisotropic soft magnetic materials, which is a key material basis for building high-performance sensors [189]. Shi et al. [190] developed a multifunctional biocompatible hydrogel based on poloxamer micelles via DLP 4D printing technology, which not only possesses good biocompatibility, but also has sensing properties that make it an ideal candidate for customized wearable health monitoring devices.

From the perspectives of structural components and printing process, Xiao et al. prepared a flexible sensor consisting of a sensing unit based on CNT/elastomer (MWCNT/EA) composites, interconnectors and amplifier circuits of silver nanowires on positive-charged substrate (Ag NWs@PCS). The sensitivity of the DLP-3D-printed MWCNT/EA sensing cell with stacked structure was 15.04 MPa^−1^, much higher than the solid counterpart with solid structure (1.62 MPa^−1^) (Fig. 10F and G). The sensitivity of the interconnectors with a serpentine configuration to external deformation is greatly suppressed compared to the straight Ag NW@PCS interconnectors. The interface between the sensing unit and the interconnect is mechanically robust due to the formation of chemical bonds under UV radiation. Fully 3D-printed 4 × 4 sensor arrays with integrated amplification circuits show their ability to detect pressure distributions [191]. Su et al. present a 4D-printed electromagnetic architecture (EMA) with ultrafast shape deformation and high-sensitivity detection. The EMA consists of 4D-printed polymer scaffolds with magnetic and conductive portions along the EMA. The EMA combines an ultrashort deformation response time as low as 7 ms and a high sensitivity detection capability of 0.4 Pa high sensitivity stress detection limit (Fig. 10A and B). By integrating the EMA with a microcontroller unit (MCU) and an external power supply, an intelligent capture device capable of sensing and responding to a variety of external stimuli is obtained, which can be used to recognize and capture target objects of a specific quality. This work realizes the efficient integration of ultra-fast shape deformation capability and ultra-low stress sensing function in 4D-printed architectural structures, which provides a feasible technological path for smart devices in the field of integrated deformation-sensing development [3].

The combination of smart material innovation and advanced structure/process makes it possible to realize multi-physical quantity sensing, intelligent response, and system-level integration on a single flexible platform. This synergistic effect greatly enhances the performance boundaries and functional diversity of flexible sensors, and these research results not only promote the development of flexible sensor technology, but also lay a solid technological foundation for their wide application in cutting-edge fields such as human body detection and environmental detection, and are expected to achieve more substantial breakthroughs in the future in terms of the optimization of the performance of the magnetically responsive materials, the improvement of the manufacturing process of flexible sensors, and the expansion of the application scenarios of their integration.

#### Functional device

Nowadays, as the core support of many fields, the optimization of the performance and fabrication process of functional devices has been a research hotspot. Especially for millimeter-scale functional devices with complex structures, breakthroughs in fabrication technology are of great importance in promoting miniaturization and integration. The rise of magnetically responsive materials has injected new vigor into the fabrication of functional devices. Although traditional 3D printing based on projection light curing can fabricate millimeter-scale complex structures, frequent switching of materials or processes is often required to achieve synergy between magnetic responsiveness and electrical conductivity in the fabrication of magnetic–electrical dual-functional devices, which leads to inefficiencies and limited precision, and becomes a key bottleneck in restricting the performance of the devices and the promotion of their applications. In recent years, through the deep integration of magneto-responsive materials and advanced printing processes, researchers have developed a variety of single-material, single-process integrated fabrication strategies, which have substantially improved the fabrication efficiency of bifunctional devices.

**Fig. 11.**
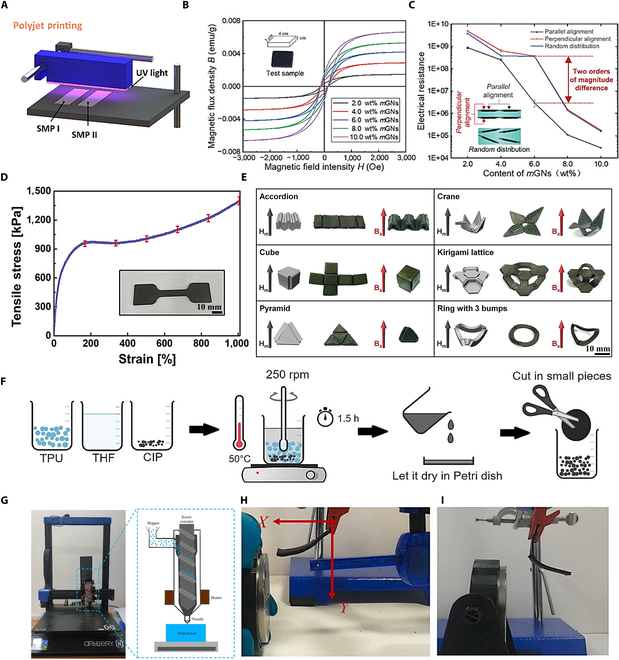
(A) SMP-based 4D printing process embedding stretchable heating circuits with fractal patterns [193]. Copyright 2021, American Chemical Society. (B) Magnetic properties of printed building blocks containing different contents of magnetic graphene nanosheets. (C) Electrical conductivity of printed building blocks containing different contents of magnetic graphene nanosheets [192]. Copyright 2024, Wiley. (D) Stress–strain curve of the 4D-printed dog-bone-shaped MSMs. (E) Various printed MSM architectures driven in magnetic fields [141]. Copyright 2023, American Chemical Society. (F) Solvent casting and (G) 4D printing for the preparation of MRE feedstocks [195]. Copyright 2025, Wiley. Stable maintenance of both morphologies is achieved by means of the synergistic effect of the Joule thermal effect and magnetic field excitation. (H) Attraction. (I) Repulsion [196]. Copyright 2023, Elsevier.

Li et al. proposed a new single-material PSL process (using chemical deposition to modify graphene nanosheets [GNs] with Fe_3_O_4_ magnetic particles and doping them into photosensitive resins to develop new bifunctional printed materials with magnetic and localized conductive properties) for the fabrication of millimeter-scale bifunctional with magnetic and localized conductive properties by electric field-assisted oriented construction of a magnetic–electrical bifunctional material (Fig. 11B and C). During the printing process, the deflection and alignment of the modified magnetic graphene nanosheets (mGN) were precisely controlled by applying an electric field, which led to the smooth formation of conductive pathways in the alignment direction of the mGN, realizing local electrical conductivity in a specific direction and ensuring that the printed structure as a whole maintains good magnetic properties. In order to verify the effectiveness of this process, they fabricated magnetically driven circuit switches and signal encoding devices, which substantially improved the fabrication efficiency and precision of millimeter-scale bifunctional devices, and strongly promoted their application in miniaturized and integrated device fabrication [192].

On the other hand, Zhang et al. innovatively proposed an SMP-based 4D printing process (Fig. 11A), which realizes multifunctional integration through structural design. Specifically, using electric field-driven micro 3D printing, stretchable heating circuits based on the fractal principle are cleverly integrated into the initial planar shape deformation structure. The overall resistance and area coverage of the circuits can be precisely adjusted by flexibly controlling the fractal order, printing parameters, and post-processing conditions, resulting in highly efficient and homogeneous heating performance and good stretchability of the structure [193].

Wajahat et al. developed an innovative 4D printing strategy for the fabrication of super-stretched MASM architectures. They fabricated the 3D structure of MSM using the DIW technique and programmable magnetization of the printed architecture using an origami-inspired magnetization method. Up to 1,000% stretchability was achieved using NdFeB-SIS composite ink (Fig. 11D). Under a highly pulsed magnetic field of 2.7 T, the magnetization distribution was designed to enable the structure to be folded into a predefined shape, and shape deformation and restoration were achieved under the action of an external magnetic field (Fig. 11E). This strategy was successfully applied to fabricate multi-finger magnetic soft grippers and soft 3D electrical switches with conductive and magnetic functions, demonstrating the potential for a wide range of applications in the field of soft actuators [141].

Functional devices serve as a fundamental component across numerous fields; consequently, the ongoing optimization of their performance and manufacturing processes remains a persistent research priority. Especially with the complex structure of millimeter-scale functional devices, its breakthrough in manufacturing technology to promote miniaturization and integration process is of great importance. The rise of magnetically responsive materials has injected new vitality into the manufacturing of functional devices. By integrating magnetically responsive materials into the manufacturing process, it is expected to give functional devices unique magnetic properties and greatly expand their functional scope. Based on 4D printing technology and other advanced manufacturing means, the application of magnetically responsive materials in functional devices provides a realization path. However, there are still many dilemmas in the use of magnetically responsive materials in the manufacture of functional devices, such as the efficiency and precision problems caused by frequent switching between multiple materials or processes, which seriously impede the large-scale application and popularization of magnetically responsive materials in the manufacture of functional devices, and innovative technologies and processes are urgently needed to break this deadlock.

### Other

In addition to the abovementioned findings, magnetically responsive materials also have potential applications in other fields. For example, in acoustic regulation, Xue’s team prepares magnetic shape memory composite honeycomb film through light-curing 4D printing and utilizes the deformation of the structure triggered by heat generation of magnetic particles under the alternating magnetic field to realize dynamic adjustment of honeycomb height, which overcomes the difficulties of traditional acoustic materials in band fixation and poor flexibility [194]. Sukarman’s innovative combination of solvent casting and 4D printing simplifies the MRE manufacturing process (Fig. 11F and G), giving the material programmable mechanical properties [195]. Lalegani Dezaki and Bodaghi [196] designed an MRE-based bistable electroactive actuator (Fig. 11H and I), which integrates a conductive SMP and MRE through 4D printing, realizing the programmed drive of the magnetic field and the self-locking position function, which substantially reduces energy consumption. Yang et al. [197] used this technology to develop geometrically reconfigurable lightweight metamaterials, breaking through the limitations of traditional mechanical metamaterial structures with solidified functions, and providing a new paradigm for adaptive cushioning interfaces and deformed space structures. These technologies integrate a magnetic–thermal force multi-field coupling drive and a bistable energy-saving design, and promote the evolution of acoustic systems, drives, and space structures toward dynamic programmability.

## Conclusion and Perspective

4D printing technology introduces the dimension of “time”, enabling the printed structure to undergo dynamic changes under external stimuli such as magnetic fields. Magnetic responsive materials have become a research focus due to their characteristics of large deformation, rapid response, and non-contact manipulation, and have important applications in fields such as biomedicine and robotics. However, current magnetically responsive materials still face challenges including limited magnetic strain and low printing precision for complex structures.

In the future, material innovation needs to develop materials with higher magnetic strain and biocompatibility, optimize the processability of magnetic shape memory materials, construct a “magnetic–electric–thermal” multi-field coupling response system, and realize the leap of deformation accuracy from the millimeter level to the micrometer level. Printing technology should realize multimaterial synergy and micro-nano scale printing, and combine calculation to optimize parameters to improve accuracy and efficiency. In terms of application, it is necessary to deepen the application in interdisciplinary fields and bring changes to fields such as intelligent manufacturing.
